# Population under Stress: The Coping Mechanisms of Lebanese Adults in Times of Intermingling Crises and Their Relationship with Stress

**DOI:** 10.1155/2024/7281288

**Published:** 2024-03-30

**Authors:** Hazem Iaali, Karine Eid, Alex El Darzi, Jennifer Joukhdar, Nour Khoury, Miran A. Jaffa

**Affiliations:** ^1^Faculty of Medicine, American University of Beirut, P.O. Box 11-0236 Riad El-Solh, Beirut 1107 2020, Lebanon; ^2^Epidemiology and Population Health Department, Faculty of Health Sciences, American University of Beirut, Beirut, Lebanon

## Abstract

The Lebanese population has faced numerous stressors due to multiple crises in the past four years. This study aims to measure the perceived stress of the Lebanese population, identify the coping mechanisms being used, and determine whether they are associated with their stress levels. A cross-sectional study of 205 individuals randomly selected from Beirut was conducted. Frequency distribution, descriptive analysis, and multivariable cumulative logit models were used to determine the associations between coping mechanisms and perceived stress. Our results indicated that 95.4% of our population had moderate to high perceived stress levels. Problem-focused coping was the most adopted mechanism and was associated with a statistically significant lower stress level, whereas avoidant coping was associated with a statistically significant higher stress level. Our study can pave the way for raising awareness on the importance of managing stress with adaptive coping mechanisms.

## 1. Background

During the past five years, Lebanon has been battling with the repercussions of intermingling crises that have overwhelmed its citizens. Political instability has been a longstanding problem in Lebanon, with a complex sectarian political system that has continuously undermined the democratic process in the country. The political movements in Lebanon are largely led by religious sects, which have reinforced clientelism and nepotism as pillars of the political regime [1]. The government has repeatedly failed in addressing the lingering economic issues of the country and implementing crucial reforms, eventually leading, in October 2019, to widespread protests against corruption. Health, wellbeing, and a decent quality of life with infrastructure reforms were at the top of the protesters' demands. Following this movement, the country entered a social and economic collapse, where schools, banks, and administrative functions were all controlled by what was happening on the ground [1]. Lebanese pound has lost more than 90% of its value, leaving half of the population below the poverty line [2, 3]. The banking sector has faced significant challenges, leading to restrictions on withdrawals and capital controls, where depositors were unable to withdraw their life savings from the banks. The economic crisis has resulted in a scarcity of crucial goods and services such as fuel, electricity, and medical supplies. This has greatly amplified the everyday hardships faced by the citizens of Lebanon, causing widespread stress and frustration. The COVID-19 pandemic, which reached Lebanon at the start of 2020, further exacerbated the instability within the country, leading to illness, job stress, and a huge burden on the fragile healthcare system [4]. Similar to what happened worldwide, the pandemic has resulted in thousands of patients in dire need of urgent medical care and hundreds of deaths [5]. The poor healthcare infrastructure, along with the banking sanctions, has left many hospitals in Lebanon with limited capacity to supply medications and even hospital beds and ventilators for their patients [1]. Moreover, the economic and social repercussions of lockdowns have greatly compounded the difficulties experienced by the population. The effectiveness of this lockdown in a country like Lebanon was debatable, as compared to other countries under lockdown. For instance, high-income countries, such as the United States, were found to experience a more efficient lockdown, with a better control of the outbreak of this pandemic [6]. Lebanon's unforeseen economic and social struggles have surely reduced the effectiveness and compliance of the lockdown and may have increased the stress of living rather than alleviating it. The Beirut blast in 2020 has escalated the dire situation, in which stored ammonium nitrate at the Beirut port exploded. The explosion has led to hundreds of thousands of lost homes, thousands of injuries, and over 200 deaths, leaving the population in a devastating state [7]. The repercussions of these cumulative crises still radiate to this day, with no foreseen solutions and no expected initiatives from the government. Humanitarian organizations have been working to aid, but the scale of the crisis has presented significant challenges [1]. More recently, Lebanon's unfortunate location in the middle of geopolitical tension within the Middle East added another layer of stress to the population. The weak political power of the country has stripped the Lebanese state of its independence. International influences, proxies of regional politics, and neighboring wars and conflicts all exacerbated the uncertainty in the fate of the Lebanese people and positioned them in a unique situation where they were subjected to a multitude of different stressors [8].

The magnitude and long-term subsequent effect of these stressors, the impact of the multifactorial and pertinent crises on the wellbeing of the Lebanese population, and the strategies that are being employed to cope with the dwindling economic and social conditions have not been fully explored yet. In spite of the gravity of the situation the Lebanese population is enduring, limited research has been performed on this subject. In particular, studies that have been conducted thus far focused primarily on selected stressors and their respective possible implications, such as the work of Maatouk et. al [9] that was centered on COVID-19 and its psychosocial correlates in Lebanon.

Accordingly, it is of great importance to gather a holistic overview of the combination of stressors in Lebanon and their effects on the population, as each stressor was shown to have different mental and physical health implications. Furthermore, it is relevant to note the association between stress and the coping styles used by the Lebanese population. In the literature, coping mechanisms and stress levels have been assessed in specific Lebanese subpopulations such as basketball players [10], university students [11], and cancer patients [12]. However, considering the Lebanese crises that were highlighted earlier, it seems reasonable to expand the scope of these studies and consider the entire Lebanese population as a population under stress. Accordingly, in this study, we aim to understand the overall effect of the crises in Lebanon on stress levels in the general Lebanese population and identify the coping mechanisms that are prevalent in this community. Our objective is to measure the perceived stress that the Lebanese population is experiencing under the effects of this unique combination of stressors. Then, we aim to delineate the coping mechanisms that are most common in this environment and how they compare to those that were reported in the literature. Last, our goal is to uncover the associations between stress levels and different coping mechanisms, using multiple stratifications of the latter, to draw useful conclusions pertaining to the way the Lebanese population is handling the crises.

## 2. Materials and Methods

### 2.1. Study Design and Sampling Strategy

We carried out a cross-sectional study, with a convenience sample of Lebanese pedestrians collected from Beirut, the capital of Lebanon. A self-administered questionnaire was distributed to Lebanese citizens who could be residing in, or outside Beirut, and data were collected between February and April of 2023. Participants were approached in a random manner and were asked for consent to answer the 10-minute questionnaire. After consenting, they were given the option to fill in the survey in their preferred language, Arabic or English, while emphasizing the anonymity and confidentiality of their responses. The inclusion criteria limited the participation in the study to Lebanese citizens only, aged 18–64, who understand English or Arabic, and have been living in Lebanon since 2020, for them to have experienced the repercussions of the aforementioned crises.

It is noteworthy to point out that Beirut is the largest commercial district in the country which attracts individuals with diverse social, economic, and demographic backgrounds. Hence, collecting data from its major areas helps in the representativeness of our sample and the generalizability of our results.

### 2.2. Concepts, Indicators, and Measurements

The surveys gathered data on the subjects' demographics, including their age, gender, citizenship, place of residency, marital status, educational level, and monthly income estimate. Information on the impact of the emerging circumstances in Lebanon was also collected. These data encompassed questions on materialistic losses and injuries from the Beirut blast, health or financial burdens resulting from the COVID-19 pandemic, and spending problems related to the Lebanese economic situation and banking sector collapse. In addition, the surveys gathered information on the participants' perceived stress and coping mechanisms by using two validated scales, which are the perceived stress scale (PSS-10) [13], which is used to measure subjective levels of stress, and the brief-Coping Orientation to Problems scale (brief-COPE) [14] questionnaire, which is used to assess different methods of coping with stressful events. The PSS-10 scale is validated in both the English and Arabic languages. Arabic PSS-10 was validated in a Lebanese subpopulation [15] and was used in this study. The rest of the questionnaire was translated by an Arabic translator, including the brief-COPE questionnaire.

The brief-COPE stratifies coping styles into three different categories: problem-focused coping, emotion-focused coping, and avoidant coping [16]. Problem-focused coping conveys the ability to deal with stressful situations or change them, assesses psychological strength, and employs a practical approach to solving problems. Emotion-focused coping refers to attempting to manage one's emotions when facing challenges. For example, self-criticism and venting are some of the themes of this coping mechanism. Avoidant coping refers to making mental or physical efforts to avoid a challenging situation. For instance, substance use and a work-centered life are some themes of avoidant coping.

The three categories were then divided into 14 subcategories of coping [17]. Active coping, informational support use, positive reframing, and planning are considered subcategories of problem-focused coping. Emotional support, venting, humor, acceptance, religion, and self-blame are subcategories of emotion-focused coping. Self-distraction, denial, substance use, and behavioral disengagement are subcategories of avoidant coping. Dividing our categories into subcategories is important to collect information on the specific adaptive and maladaptive coping techniques that the participants used.

Furthermore, we gathered data pertaining to coping mechanisms that the brief-COPE questionnaire omitted or did not directly address, such as the use of alcoholic beverages, smoking, illicit drugs, sexual activity, medications, exercise, and seeking professional mental health support [18, 19].

### 2.3. Sample Size Calculation

The sample size was calculated from a regression angle with a low to medium anticipated effect size of 0.15, a statistical power level of 0.8, 20 predictors, and a probability level of 0.05. This specification resulted in a total sample size of 156. To account for missing data and nonresponse, we inflated the sample size to 205 individuals randomly selected from Beirut, the capital of Lebanon.

### 2.4. Plan of Analysis

We started our analysis by conducting frequency and descriptive analyses that served two purposes: data cleaning and data summary. The demographic characteristics of our respondents are presented in a table with their respective counts and percentages. Data on crisis-related difficulties and additional coping mechanisms were also presented as graphical displays with their respective counts and percentages. The PSS-10 scale is a 5-point Likert scale (0–4) with 10 items. The total score for the perceived stress level was computed by adding all the scores included in this section, with the lowest total score being 0 and the highest total score being 40. Questions 4, 5, 7, and 8 were recoded so that a score of 0 becomes 4, a score of 1 becomes 3, a score of 2 remains the same, a score of 3 becomes 1, and a score of 4 becomes 0. This recoding was done to reflect higher stress since these questions were phrased in a way that represented a lower stress level. Accordingly, the higher the score for stress, the higher the stress level. The stress scores were subsequently divided into three sections (low, moderate, and high stress) based on a cutoff for each category, and the proportion of each category was calculated [20]. The cutoffs used were (0–13) for low stress, (14–26) for moderate stress, and (27–40) for high stress. As for the coping mechanisms, as noted earlier, there are three coping styles studied: problem-focused coping, emotion-focused coping, and avoidant coping, which were further stratified into 14 subcategories. Coping scores are calculated for the 3 categories and 14 subcategories as average scores (sum of item scores divided by number of items), indicating how much the participant engaged in each coping style. The additional coping mechanisms were recoded as yes or no.

Stratified analyses comparing the levels of stress (low, moderate, and high) across the different levels of each of the demographic characteristics were carried out using the exact Fisher test for all the variables except for age, where one-way analysis of variance (one-way ANOVA) was employed. The distribution of the levels of stress across the different categories of each demographic characteristic was presented in terms of frequencies and percentages.

Similarly, stratified analysis was also conducted on each of the 3 coping mechanisms (problem focused, emotion focused, and avoidant coping) and demographic characteristics, whereby the mean and standard deviation (SD) of each coping mechanism were reported for every level of the demographic variables. Associations were determined using one-way ANOVA, followed by post hoc analysis using Bonferroni multiple comparisons for all the demographic variables, except for age, where Pearson's correlation and corresponding significance were reported.

A multivariable cumulative logit model with proportional odds property for ordinal responses was conducted on the outcome of interest, perceived stress (low, medium, and high), and the main predictors, which were the three coping categories each measured on a continuous scale computed as indicated earlier (problem-focused coping, emotion-focused coping, and avoidant coping), additional coping (yes/no), and crisis-related difficulties. Additional analysis on the ordinal outcome perceived stress, stratified as low, medium, and high, was also carried out using the subcategories of coping as main predictors.

### 2.5. Ethical Considerations

The participants were given a 2-meter distance to fill out their surveys, which were returned in a sealed envelope to ensure confidentiality. The study participants were fully informed about the scope, aims, and contents of the study before giving their consent. Voluntary participation was emphasized, and no incentives were offered. Furthermore, a brochure was given to all participants indicating the signs of stress and the available free mental health and substance abuse services. The IRB department of the American University of Beirut approved our research project (IRB ID is SBS-2022-0341).

## 3. Results

### 3.1. Demographics

Our final sample consisted of 205 participants, which is almost equally distributed between females (50%) and males (46%), with other (1%), no response (3%), and a mean age of 36.9 (standard deviation of 13.58). Almost half of the participants reported being single (50.2%), and 39.5% were married. A big majority (68.3%) indicated that they have a university degree, are employed (63.9%), and have a monthly income ranging between $101 and $300 (23.4%) (Table 1).

### 3.2. Effects of the Intermingling Crisis on the Lives of the Lebanese Population

**Figure 1:**
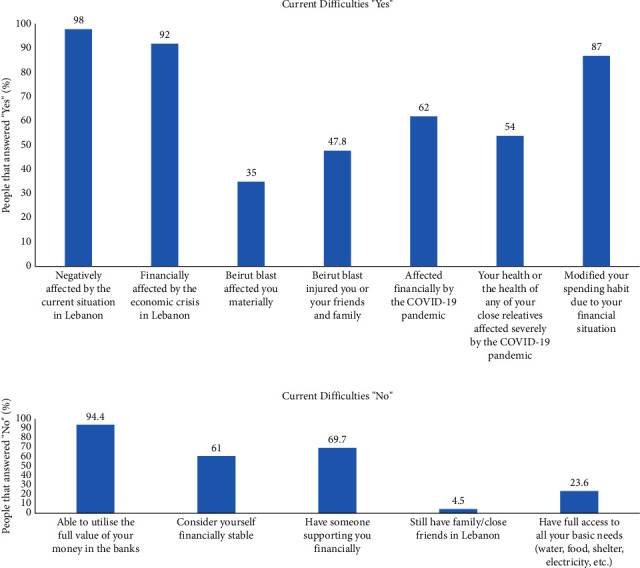
(a) Percentages of those who responded “yes” to questions about the negative effect of the intermingling crises in Lebanon on their lives. (b) Percentages of those who responded “no” to having access to certain support and/or basic needs.

Questions that assessed the effect of the crisis on the Lebanese population are all shown in Figures 1(a) and 1(b), along with the frequency distribution of the responses to each of the questions expressed in terms of percentages of positive answers (Figure 1(a)) and negative answers (Figure 1(b)). Our results (Figure 1(a)) showed that the vast majority of the participants (98%) were negatively affected by the current situation in Lebanon, financially affected by the economic crisis in Lebanon (92%), and had to modify their spending habits due to the financial situation (87%). As for the effect of the Beirut blast, 47.8% of the participants reported that they were themselves or had family members and/or friends injured by the blast, and 35% of them reported that they were materially affected by it. With respect to the effect of COVID-19, more than half of the participants indicated that they were financially affected by this pandemic (62%) and/or had a health-related effect due to this pandemic (54%). With respect to the questions pertaining to access to basic needs and financial stability/support depicted in Figure 1(b), the majority of the participants reported that they were unable to utilize the full value of their savings in the Lebanese banks (94.4%) and that they had no one that was providing them with any financial support (69.7%), nor were they considering themselves financially stable (61%). Moreover, 23.6% indicated that they have no access to basic living needs such as water, food, shelter, and electricity, and 4.5% reported that they were left with no family and no close friends who are still living in Lebanon (Figure 1(b)).

### 3.3. Frequency Distributions of the Perceived Stress Scale and Coping Mechanisms on the Lebanese Population

**Figure 2:**
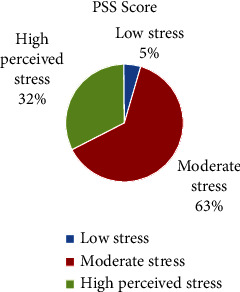
The PSS score of participants.

The frequency distribution of the perceived stress scale (PSS Score) classified as high, moderate, and low is depicted in Figure 2. Our results showed that 32% of the study population were classified as being under high stress, 63% under moderate stress, and only 5% under low stress.

When stratified by demographic characteristics (Table 2), our results did not show a significant difference in the three levels of stress and age (Fisher's exact test *P*=0.790), gender (Fisher's exact test *P*=0.608), level of education (Fisher's exact test *P*=0.394), employment status (Fisher's exact test *P*=0.608), and monthly income (Fisher's exact test *P*=0.608).

**Figure 3:**
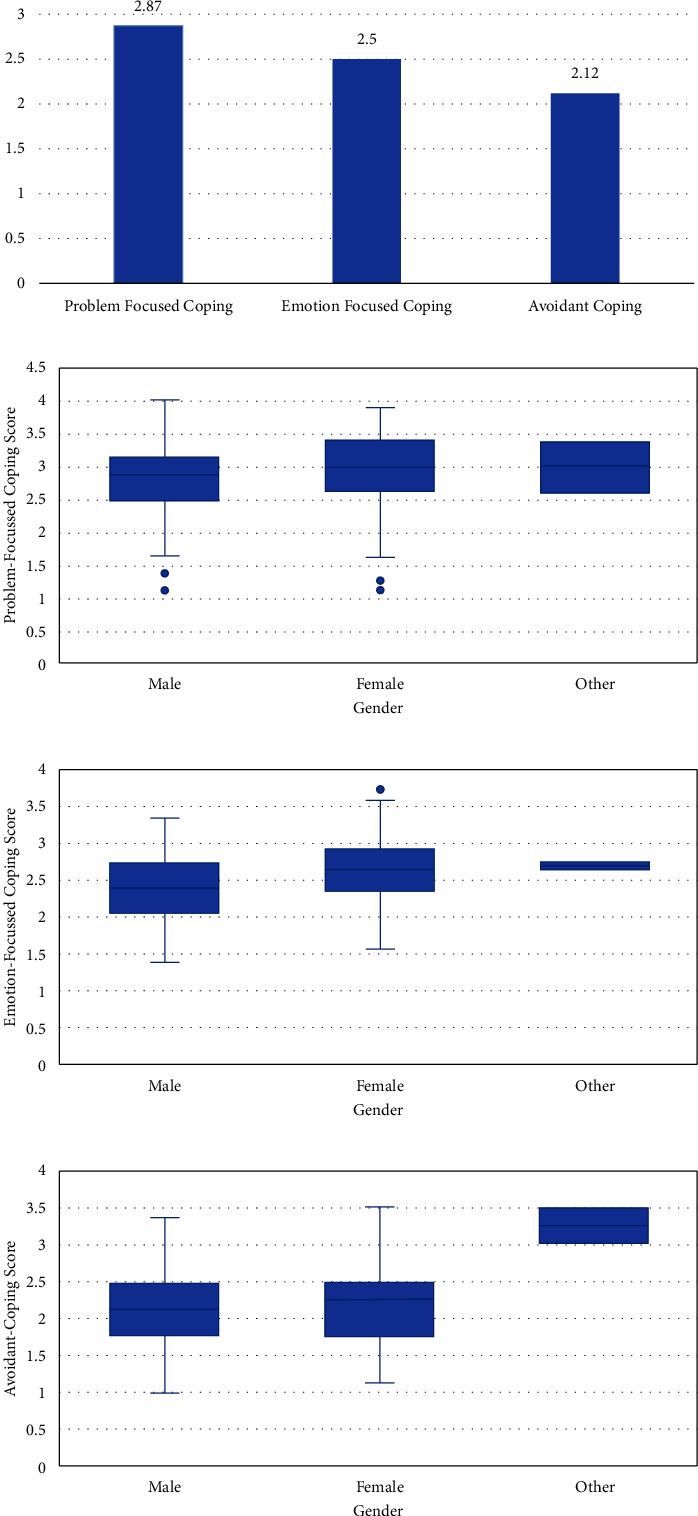
(a) Median scores for the three categories of coping mechanisms. (b) Problem-focused coping vs. gender (ANOVA *P*=0.517). (c) Emotion-focused coping vs. gender (ANOVA *P*=0.001). (d) Avoidant coping vs. gender (ANOVA *P*=0.007).

The summary statistics of the brief-COPE scores categorized into “problem-focused coping,” “emotion-focused coping,” and “avoidant coping” scores presented in terms of median scores are displayed in Figure 3(a). Our results indicated that the problem-focused coping score had the highest median score of 2.87 among the study participants, while the emotion-focused coping score median was 2.5, and the avoidant-focused coping score median was 2.12. When stratified by gender, there was no statistically significant difference in problem-focused coping between the different genders (ANOVA *P*=0.517) (Figure 3(b)). However, there was a significant difference between genders in emotion-focused coping (ANOVA *P*=0.001) as shown in Figure 3(c). In particular, females had higher emotion-focused coping scores on average than males (mean difference = 0.233, Bonferroni adjusted *P* < 0.001). As for avoidant coping, a significant difference in gender was also detected in this coping strategy (ANOVA *P*=0.007) as depicted in Figure 3(d). In this regard, participants who identified their gender as “other” had a higher score of avoidant coping than males (mean difference = 1.14, Bonferroni adjusted *P*=0.005), and females (mean difference = 1.085, Bonferroni adjusted *P*=0.009). Accordingly, as per our results, females appear to be relying mostly on emotion-focused coping, while other genders are more dependent on avoidant coping. This result is also presented in Table 3.

As for the remaining demographic characteristics (Table 3), our stratified analysis also revealed a significant link between education and emotion coping (ANOVA *P*=0.004) and with avoidant coping (ANOVA *P*=0.044). Interestingly, our data showed that participants with the highest level of education (doctorate degree) were the ones who relied the least on any of these coping mechanisms, as indicated by their lowest mean scores of 2.354 and 1.843, compared to individuals with lower degrees of education (less than high school diploma and vocational degree). Similarly, participants who reported the monthly income of more than 1000 dollars were the ones who least depended on emotion coping (mean score 2.315), the only significant coping mechanisms that differed between the income levels (ANOVA *P*=0.002), compared to those with much lower income who relied more on emotion coping (mean score 2.825). Employment status and age did not show any relationship with the different coping mechanisms (ANOVA *P* > 0.05). Results of the stratified analysis of coping mechanisms based on demographic characteristics are all reported in Table 3.

**Figure 4:**
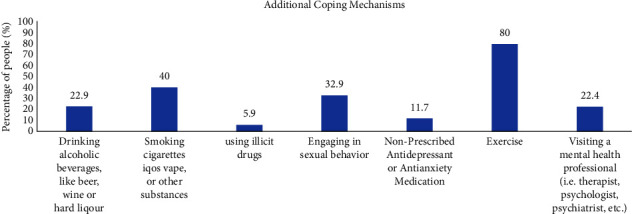
The additional coping mechanisms adopted by participants.

Questions concerning additional coping mechanisms detailed in Figure 4 revealed that the majority of participants (80%) are using exercise as a coping mechanism to alleviate their stress; some are relying on smoking (40%) and/or alcohol drinking (22.9%), resorting to sexual activities (32.9%), seeking mental health support (22.4%), using nonprescribed antidepressant or antianxiety medication (11.7%), and using illicit drugs (5.9%).

**Figure 5:**
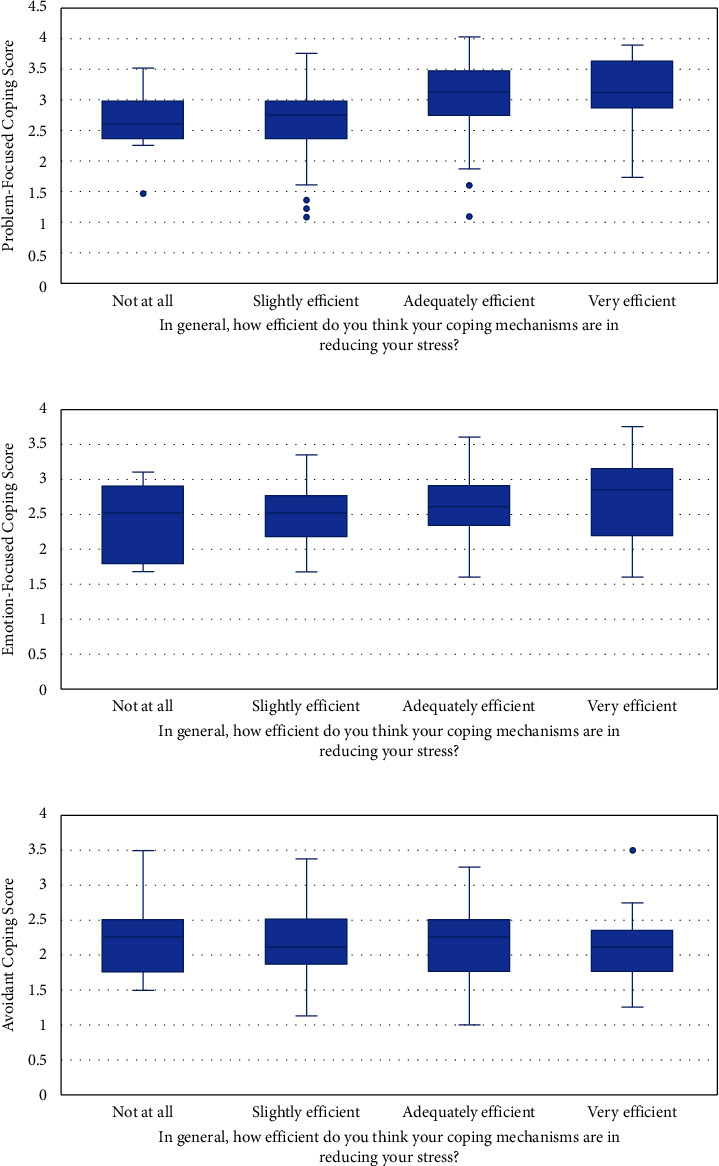
(a) Median scores for problem-focused coping stratified by the levels of satisfaction in its effectiveness in reducing stress. (b) Median scores for emotion-focused coping stratified by the levels of satisfaction in its effectiveness in reducing stress. (c) Median scores for avoidant coping stratified by the levels of satisfaction in its effectiveness in reducing stress.

**Figure 6:**
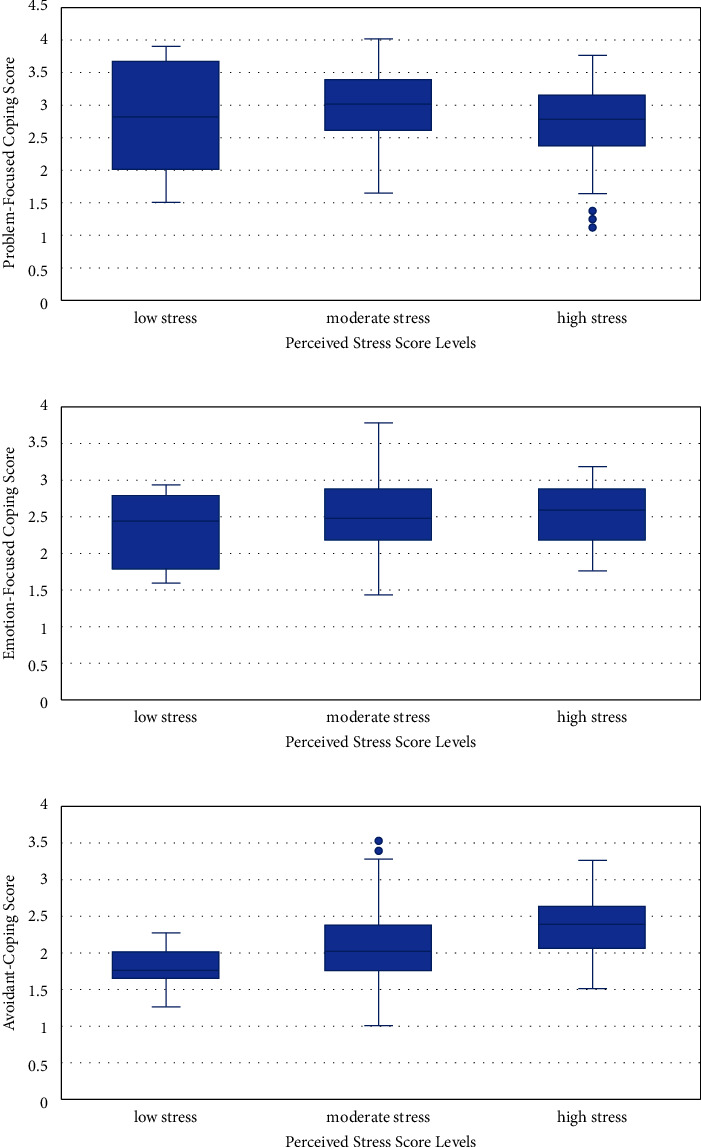
(a) Median scores for problem-focused coping stratified by the levels of stress (ANOVA *P*=0.022). (b) Median scores for emotion-focused coping stratified by the levels of stress (ANOVA *P*=0.49). (c) Median scores for avoidant coping stratified by the levels of stress (ANOVA *P* < 0.001).

As for the effectiveness of the adopted coping mechanism, 5.5% indicated that it is not helping them at all in reducing their stress, 35.2% reported that it was slightly efficient, 51.1% indicated that it was adequately efficient, and 8.2% reported that their coping mechanism was very efficient (these statistics are reported here but not displayed in a table or figure). When the coping mechanisms were stratified by their effectiveness with regard to stress, the problem-focused coping mechanisms were found to be mostly adequate to very efficient in reducing stress (mean score = 3.158, median score = 3.125) (Figure 5(a)), emotion-focused was reported as very efficient (mean score = 2.722, median score = 2.833) (Figure 5(b)), and avoidant coping was reported as not efficient (mean score = 2.236, median score = 2.25) to adequately efficient (mean score = 2.133, median score = 2.25) (Figure 5(c)). When stratified by the levels of stress, problem-focused and emotion-focused were most utilized among those with moderate stress (respective mean score problem-focused = 2.959, median score = 3, ANOVA *P*=0.022 (Figure 6(a)); mean score emotion-focused = 2.518, median score = 2.458, ANOVA *P*=0.49 (Figure 6(b))), while avoidant coping was highly utilized among those with high stress (mean score = 2.381, median score = 2.375, ANOVA *P* < 0.001 (Figure 6(c))).

### 3.4. Adjusted Associations between Coping Mechanisms, Crisis-Related Difficulties, and Stress Levels

A multivariable cumulative logit model with proportional odds property for ordinal responses was conducted on stress as an outcome with ordered categories of low, medium, and high levels. The factors considered in this analysis included the scores of the coping categories (problem-focused, emotion-focused, and avoidant), current difficulties, and additional coping questions (Table 4). None of the demographic factors were significantly associated with stress and were removed from the model. In addition, the 14 subcategories of the problem-focused, emotion-focused, and avoidant were also considered as the main predictors of stress (Table 5), along with the crisis-related difficulties and the additional coping questions. Given that the proportionality assumption of odds across the different cuts of the ordinal outcome was met (*P* value >0.05), a single odds ratio (OR) was generated for the different cuts of the outcome stress (low, medium, and high). Results of the multivariable ordinal logistic regression are presented in terms of adjusted ORs and confidence intervals (CI) and corresponding *P* values displayed in Tables 4 and 5.

### 3.5. Effect of the Three Categories of Coping Mechanisms and Additional Coping on Stress

Our results showed that the odds of having higher levels of stress were 1/0.502 = 1.99 times lower for those who had increased problem-focused coping mechanism scores. That is, using the problem-focused coping mechanism is associated with a reduced magnitude of stress (*P*=0.032) whereas the odds of having higher levels of stress were 5.8 times higher for those who had increased levels of avoidant coping mechanism scores indicating that relying on the avoidant coping mechanism is associated with an increased magnitude of stress (*P* < 0.0001). With respect to the effect of additional coping mechanisms on stress, our results showed that the odds of having high levels of stress for those who use illicit drugs and those who exercise were 1/0.103 = 9.7 and 1/0.308 = 3.24 times lower than those who do not use illicit drugs and do not exercise, respectively. That is, illicit drugs (*P*=0.005) and exercise (*P*=0.013) were shown to be associated with a reduced magnitude of stress.

### 3.6. Effects of Current Crisis-Related Difficulties

Regarding the effect of difficulties on stress (Table 4), our results showed that the odds of having higher levels of stress for those who consider themselves financially stable were 1/0.29 = 3.44 times lower than those who do not consider themselves financially stable, reflecting that financial stability is associated with a reduced magnitude of stress (*P*=0.002). Similarly, the odds of having higher levels of stress for those who have someone supporting them financially and those who have full access to their basic needs were 1/0.435 = 2.3 and 1/0.392 = 2.55 times lower than for those who do not have someone supporting them financially or have full access to their basic needs. That is, having someone to support the participants financially (*P*=0.043) and having full access to one's basic needs (*P*=0.025) were associated with a reduced magnitude of stress.

In summary, our results (Table 4) indicated that participants who used illicit drugs, exercised, and used problem-focused coping mechanisms were less likely to have high-stress levels. Similarly, those who consider themselves financially stable, have someone supporting them financially, or have access to their basic needs exhibited lower odds of high stress. Meanwhile, participants who used avoidant coping mechanisms were more likely to associate with high stress levels.

A second multivariable cumulative logit model with proportional odds property for ordinal responses was performed. The outcome was stress (low, medium, and high), and the predictors of interest were the 14 coping subcategories of problem-focused, emotion-focused, and avoidant coping mechanisms, current difficulties, income, and additional coping questions (Table 5).

### 3.7. Effect of the 14 Subcategories of Coping Mechanisms and Additional Coping on Stress

Our results showed that the odds of having high levels of stress were 1/0.584 = 1.7 and 1/0.502 = 1.99 times lower for those who had increased scores of positive reframing (*P*=0.023) and acceptance (*P*=0.01) as coping mechanisms, respectively (Table 5). However, the odds of high stress were 1.96 and 2.44 times higher for those with increased scores of venting (*P*=0.016) and self-blame (*P* < 0.001) as coping mechanisms. The remaining subcategories of coping mechanisms were not significantly associated with stress levels. With respect to the additional coping strategies, exercise was the only coping mechanism that showed a significant association with stress (*P*=0.018) whereby the odds of high stress were 1/0.339 = 2.95 times lower for those who exercise versus those who do not exercise.

In addition, our results (Table 5) also showed that the odds of high stress for those who have full access to their basic needs were 1/0.264 = 3.78 times lower than those who do not have full access to their basic needs (*P*=0.001) and 5.97 times higher among those whose monthly income is less than 50$ compared to those whose monthly income is greater than 1000$ (*P*=0.028).

Accordingly, our results (Table 5) showed that participants with positive reframing and acceptance as coping mechanisms, those who exercise as an additional coping mechanism, and those who have full access to their basic needs were less likely to have high stress levels. Meanwhile, participants who indicated that they used venting and self-blame as coping mechanisms were more likely to have high stress levels. Similarly, individuals whose monthly income was less than $50 were 5.9 times more likely to have higher stress levels than those whose income was above $1,000.

## 4. Discussion

We present a novel study that assesses the effect of different coping mechanisms on the magnitude of stress in a unique population that is enduring a multitude of different crises. Furthermore, our study unravels the different stressors and coping factors that have been exacerbating or ameliorating the levels of stress.

### 4.1. Stress Levels in Times of Crisis

Our survey results indicate that 95% of the sampled Lebanese adults perceive their stress levels to be moderate to high. This proportion highlights the considerable effect that the cumulative crises have had on the Lebanese population in terms of perceived stress. Other studies conducted in the context of the COVID-19 pandemic and the Beirut Blast have reported lower proportions of stress in their samples. For example, one study investigating the perceived stress levels of Lebanese subpopulations concluded that 69% of medical students and 58.1% of healthcare workers reported high to moderate stress [18]. A possible explanation for the discrepancy in stress levels is our more recent sampling, which may have encompassed the effects of the escalating financial crises and the unparalleled inflation rates [21]. In addition, we speculate that healthcare workers and medical students may possess greater knowledge on how to manage their stress in challenging times and will thus report less perceived stress. More research should be undertaken to address this claim.

### 4.2. Gender Stratification of the Brief-COPE

In our stratification of the brief-COPE results, there was no statistically significant difference in problem-focused coping scores between the three gender categories. However, a significant difference was found in emotion-focused coping between males and females, with females relying more on this type of coping. Cumulative findings in the literature show variations in how the two genders utilize coping styles, and our finding that females use more emotion-focused coping is consistent with the literature [22]. More recently, female college students and general practitioners were found to rely more on emotion-focused coping, including self-distraction, emotional support, instrumental support, self-blame, and venting, compared with males [23, 24]. A possible explanation for the different coping styles of the genders arises from the social inequalities that they may face, which may occur under similar stressors. In this regard, a study which highlighted the gender-specific favoritism in the scientific field reported that, despite similar environmental stressors, women were required to have significantly higher academic achievements and stronger interpersonal relationships to obtain the same position as their male counterparts [25]. Various underlying dissimilarities may explain why men and women develop different coping mechanisms. Moreover, when gender differences in response enforcement mechanisms were examined, women were found to be motivated by social pressures and men by financial stressors, thus offering another explanation of the intrinsic disparate motivators due to societal expectations [26].

Only 1% of our participants reported having a gender other than male or female. It is interesting to note that, using stratification, this category was found to depend more on avoidant coping than either males or females, with significant differences in their respective avoidant coping scores. However, the significance of the results should be interpreted with caution given the small proportion of other genders in our study. The literature does not highlight valuable stratified data on the brief-COPE of individuals with a fluid sexual identity; thus, no reliable conclusions can be drawn on the coping styles used by these individuals, and further studies that delve deeper into the gender effect on coping with more detailed stratifications of gender should be undertaken.

### 4.3. Association between Coping Mechanisms and Stress Levels

Our first multivariable cumulative logit model revealed statistically significant associations between stress and two of our three coping categories. Problem-focused coping was found to be associated with a lower stress level, whereas avoidant coping was found to be associated with a higher level of stress. This finding is expected since similar patterns were reported in multiple populations subjected to different types of stressors [27, 28]. Problem-focused coping was found to be associated with a better quality of life for participants in a population in the United States in the context of COVID-19 as a stressor [29]. Higher stress levels were associated with a greater reliance on avoidance-coping strategies in a cross section of a Catalonian population [24]. Given the high levels of stress in the Lebanese population, we expected a higher proportion of avoidant coping, which is considered maladaptive in nature. However, our study uncovered higher rates of problem-focused coping among respondents, which suggests that the Lebanese population is already adaptively coping with the crises by using coping mechanisms associated with lower stress levels. This suggests that other factors other than the type of coping strategies used are contributing to the elevated stress levels in the Lebanese population.

Our study investigated other coping mechanisms besides the ones mentioned in the COPE-questionnaire, which is part of our effort to shed light on the effects of some of the most harmful and beneficial coping styles. Our results suggest that some styles of avoidant coping, mainly substance abuse and the use of illicit drugs, were found to be associated with lower stress levels. Since our survey measured perceived stress levels, it is plausible that individuals experiencing intoxication may feel as though they are alleviated in the short run. This finding further emphasizes the need for awareness of the long-term effects of substance abuse on stress levels and mental or physical health. Even though the brief-COPE classifies substance abuse as an avoidant coping mechanism, one article describes the continuous use of avoidant coping styles in substance abusers as contributing to an overall increase in stress in the long run [30]. Another article highlights the dangers of substance abuse shown clearly in adolescents, as substance abusers have a much higher rate of hospital visits than their sober counterparts, as well as higher risk-taking behaviors that can result in higher rates of car accidents, sexually transmitted disorders, and even trauma [31]. Thus, efforts should be made in raising awareness of the dangers of illicit drug use in Lebanon as well as providing effective alternative coping mechanisms for individuals who want to decrease their stress levels.

Our second multivariable cumulative logit model showed that using a positive reframing strategy is associated with a lower stress level. Positive reframing is a subsection of emotion-focused coping whereby individuals shift their mindset to more positive means in challenging situations. Multiple studies have highlighted the benefits of positive reframing, including higher rates of self-compassion and posttraumatic growth [32]. Therefore, our result is consistent with the literature's findings that positive reframing is an effective coping mechanism in times of stress. In addition, exercise was significantly associated with a lower stress level. Numerous studies emphasized the multitude of benefits of exercising, including decreasing stress levels and having positive effects on both physical and mental health. Sharon-David and Tenenbaum [33] have performed a systematic review on the effectiveness of physical activity on mental and physical health, and it was found that exercising three times a week for fifteen minutes showed a significant reduction in stress levels. Furthermore, our survey results indicate that a higher income was correlated with less stress in Lebanese individuals, which is understandable as low-income families have a harder time securing basic necessities, which can be highly stress-inducing. One article reported that higher income alleviated stress levels among Lebanese adults, while lower income was associated with increased levels of stressors due to greater problems with health, social ties, and employment [34]. Our results are consistent with other studies that assessed how these different subcategories correlate with stress.

Despite of the availability of public services which include coverage for illness, disability, and protection of women's right, Lebanon still lacks social protection policies and fair labor laws that secure equal access to these services, as evidenced by the underprivileged populations benefiting the least from these public services [35]. This inequality was exacerbated during COVID-19, due to the load of sick and underprivileged patients, which eventually increased the stress on the population and the strain on the volatile Lebanese healthcare system. A similar scenario was demonstrated by Li et al, whereby high-income households benefited more from government subsidies, although those with lower incomes were more vulnerable to the effects of COVID-19 [36].

Our survey results indicate that venting and self-blame, which are subsets of emotion-focused coping, are positively correlated with stress. Venting is defined as “expressing negative feelings,” while self-blame is defined as “attributing stress to oneself”; within the literature, both of these coping mechanisms were found to be maladaptive in nature and have been associated with higher levels of stress [37].

## 5. Limitations

The main limitation of our study is that our outcome which is perceived stress is a reported finding at one point in time and cannot represent the stress levels of our population over the entire period of the crisis. This is due to the cross-sectional nature of our study, which also limits the inferences that can be drawn. For instance, data cannot be used to extract cause-effect relationships but can still show possible associations. Moreover, being self-reported, the stress levels and coping mechanisms are subjective and susceptible to bias. Response bias due to potential misunderstanding, social desirability bias, which can be present even in anonymous surveys, or recall bias as participants rely on their memories, could possibly play a role. In addition, since the sample was limited to Beirut, the results cannot be generalized to the entire Lebanese population, especially since different regions are currently experiencing different stressors with varying magnitudes.

## 6. Implications

The high levels of stress found in the Lebanese population necessitate a public health intervention, considering the multitude of health implications associated with chronically elevated stress levels [13]. Moreover, maladaptive coping styles are associated with several types of psychological distress, including depression, anxiety, and posttraumatic stress disorder [28]. Therefore, managing stress levels using adaptive coping styles should be encouraged, especially since numerous studies have shown that educating people on adaptive coping mechanisms can help navigate life's challenges and improve mental health in subpopulations under stress [38, 39]. Our results can serve as a pilot study, highlighting the effects of the escalating situation in Lebanon on its population and guiding further mental and public health research. A large-scale public health campaign that exposes the population to the principles of problem-focused coping can help reduce the mental burden of the crises. Furthermore, social awareness of adaptive versus maladaptive coping should be raised in a variety of social settings, including schools, workspaces, and common public spaces. Last, it is important to raise awareness of the free resources available for the Lebanese population to manage their stress, such as visiting counseling centers to receive professional care or visiting substance abuse centers if needed.

## Figures and Tables

**Table 1:** Demographics of participants.

Variables	Number of responses (*N*)	Percentage of responses (%)
Age		
Age response	188	91.7
Missing	17	8.3
Mean age ± SD	36.94 ± 13.58	
Gender		
Male	94	45.9
Female	102	49.8
Other	2	1
Missing	7	3.4
Highest level of education		
No degree	3	1.5
Less than a high school diploma	5	2.4
High school diploma or equivalent degree	19	9.3
Vocational degree (BT, TS, etc.)	19	9.3
University degree (bachelor's degree, master's degree)	140	68.3
Doctorate degree (Phd, MD, etc.)	12	5.9
Missing	7	3.4
Current employment status		
Freelancer	32	15.6
Employed	131	63.9
Not employed, currently a student	18	8.8
Not employed, looking for work	8	3.9
Not employed, not looking for work	6	2.9
Retired	2	1
Disabled, not able to work	2	1
Missing	6	2.9
Monthly income		
Less than $50 per month	12	5.9
Between $50 and $100 per month	24	11.7
Between $101 and $300 per month	48	23.4
Between $301 and $500 per month	36	17.6
Between $501 and $1000 per month	34	16.6
More than $1000 per month	41	20
Missing	10	4.9

**Table 2:** Perceived stress levels stratified by demographic characteristics showing frequency and percentages of stress within the levels of the demographic variables.

Variables	Stress *N* (row valid percent)			*P* value^§^
	Low	Moderate	High	
Age				
Age response *N* (%)	8 (88.88)	118 (95.16)	58 (90.62)	0.790^£^
Mean age ± SD	34.63 ± 15.72	36.42 ± 13.13	37.57 ± 14.32	
Gender				
Male	5 (5.5)	60 (65.9)	26 (28.6)	0.608
Female	3 (3.0)	63 (62.4)	35 (34.7)	
Other	0 (0.0)	50 (0.8)	50 (1.6)	
Highest level of education				
No degree	0 (0.0)	1 (50.0)	1 (50.0)	0.394
Less than a high school diploma	0 (0.0)	5 (100)	0 (0.0)	
High school diploma or equivalent degree	0 (0.0)	11 (57.9)	8 (42.1)	
Vocational degree (BT, TS, etc.)	0 (0.0)	10 (58.8)	7 (41.2)	
University degree (bachelor's degree, master's degree)	6 (4.3)	91 (65.0)	43 (30.7)	
Doctorate degree (Phd, MD, etc.)	2 (18.2)	6 (54.5)	3 (27.3)	
Current employment status				
Freelancer	1 (3.1)	15 (46.9)	16 (50.0)	0.219
Employed	6 (4.7)	88 (69.3)	33 (26.0)	
Not employed, currently a student	1 (5.6)	12 (66.7)	5 (27.8)	
Not employed, looking for work	0 (0.0)	4 (50.0)	4 (50.0)	
Not employed, not looking for work	0 (0.0)	4 (66.7)	2 (33.3)	
Retired	0 (0.0)	0 (0.0)	2 (100)	
Disabled, not able to work	0 (0.0)	1 (50.0)	1 (50.0)	
Monthly income				
Less than $50 per month	0 (0.0)	5 (41.7)	7 (58.3)	0.469
Between $50 and $100 per month	0 (0.0)	17 (70.8)	7 (29.2)	
Between $101 and $300 per month	1 (2.1)	29 (60.4)	18 (37.5)	
Between $301 and $500 per month	2 (5.9)	22 (64.7)	10 (29.4)	
Between $501 and $1000 per month	2 (5.9)	24 (70.6)	8 (23.5)	
More than $1000 per month	4 (10%)	25 (62.5)	11 (27.5)	

^§^Exact Fisher test was used to generate the *P* values. ^£^ANOVA test was used to generate the *P* value for the association between age and stress categories.

**Table 3:** Coping mechanisms stratified by demographic characteristics showing mean ± standard deviation (SD) of the coping score within the levels of the demographic variables and *P* values^**§**^.

Variables	Coping mechanisms		
	Problem focused	Emotion focused	Avoidant coping
Age			
Pearson's correlation between age and coping (*P* value for Pearson's correlation)^¥^	−0.101 (0.180)	−0.095 (0.202)	0.094 (0.210)
Gender (*P* value^§^)	(0.517)	(0.001)	(0.007)
Male	2.851 ± 0.585	2.386 ± 0.462	2.109 ± 0.544
Female	2.945 ± 0.562	2.625 ± 0.421	2.164 ± 0.465
Other	3.0 ± 0.530	2.708 ± 0.058	3.250 ± 0.353
Highest level of education (*P* value^§^)	(0.055)	(0.004)	(0.044)
No degree	3.187 ± 0.795	2.750 ± 0.471	2.375 ± 0.000
Less than a high school diploma	3.125 ± 0.000	3.083 ± 0.166	2.000 ± 0.433
High school diploma or equivalent degree	2.573 ± 0.869	2.568 ± 0.447	2.360 ± 0.607
Vocational degree (BT, TS, etc.)	3.139 ± 0.485	2.676 ± 0.338	2.404 ± 0.415
University degree (bachelor's degree, master's degree)	2.893 ± 0.536	2.483 ± 0.45716	2.124 ± 0.517
Doctorate degree (Phd, MD, etc.)	2.729 ± 0.558	2.354 ± 0.466	1.843 ± 0.406
Current employment status (*P* value^§^)	(0.329)	(0.589)	(0.418)
Freelancer	2.892 ± 0.461	2.431 ± 0.4309	2.232 ± 0.528
Employed	2.905 ± 0.577	2.542 ± 0.4760	2.137 ± 0.527
Not employed, currently a student	2.895 ± 0.462	2.472 ± 0.386	1.993 ± 0.462
Not employed, looking for work	2.906 ± 0.667	2.458 ± 0.274	2.484 ± 0.580
Not employed, not looking for work	2.583 ± 1.032	2.555 ± 0.569	2.145 ± 0.365
Retired	2.437 ± 0.795	2.250 ± 0.235	2.375 ± 0.530
Disabled, not able to work	2.125 ± 1.414	2.791 ± 0.530	2.062 ± 0.088
Monthly income (*P* value^§^)	(0.545)	(0.002)	(0.521)
Less than $50 per month	2.738 ± 0.759	2.545 ± 0.415	2.318 ± 0.341
Between $50 and $100 per month	2.994 ± 0.597	2.825 ± 0.301	2.148 ± 0.541
Between $101 and $300 per month	2.809 ± 0.641	2.470 ± 0.434	2.202 ± 0.496
Between $301 and $500 per month	2.943 ± 0.486	2.553 ± 0.444	2.083 ± 0.527
Between $501 and $1000 per month	2.996 ± 0.431	2.570 ± 0.392	2.218 ± 0.444
More than $1000 per month	2.753 ± 0.661	2.315 ± 0.532	2.055 ± 0.584

^§^ANOVA test was used to generate the *P* values. ^¥^*P* value for Pearson's correlation between age and each coping mechanism.

**Table 4:** Multivariable cumulative logit model with proportional odds property for ordinal responses, where the outcome is stress (low, medium, and high), and the predictors are the coping categories, current difficulties, and additional coping questions.

Variable	Adjusted odds ratio	95% confidence interval odds ratio adjusted	*P* value
Problem_Focused_coping_score	0.502	(0.267, 0.944)	0.0320^*∗*^
Avoidant_coping_score	5.807	(2.624, 12.846)	<0.0001^*∗*^
Drink alcoholic beverages	2.038	(0.871, 4.759)	0.1000
Smoke cigarettes, iqos, vape, or other substances	0.836	(0.381, 1.831)	0.6540
Use illicit drugs	0.103	(0.021, 0.495)	0.0050^*∗*^
Engage in sexual behavior	1.254	(0.567, 2.768)	0.5760
Take unprescribed antidepressants or antianxiety medications	0.663	(0.177, 2.469)	0.5400
Exercise	0.308	(0.121, 0.780)	0.0130^*∗*^
Visiting a mental health professional	1.249	(0.518, 3.01)	0.6200
Negatively affected by the current situation in Lebanon	8.215	(0.657, 102.617)	0.1020
Consider yourself financially stable	0.29	(0.131, 0.639)	0.0020^*∗*^
Have someone supporting you financially	0.435	(0.193, 0.975)	0.0430^*∗*^
Have full access to all your basic needs	0.392	(0.172, 0.890)	0.0250^*∗*^

^*∗*^Significant *P* values (*P* < 0.05).

**Table 5:** Multivariable cumulative logit model with proportional odds property for ordinal responses, where the outcome is stress (low, medium, and high), and the predictors are the coping subcategories, current difficulties, income, and additional coping questions.

Variable	Adjusted odds ratio	95% confidence interval odds ratio adjusted	*P* value
Positive reframing	0.584	(0.368, 0.930)	0.0230^*∗*^
Venting	1.96	(1.137, 3.377)	0.0160^*∗*^
Acceptance	0.502	(0.297, 0.850)	0.0100^*∗*^
Self-blame	2.494	(1.540, 4.040)	<0.0005^*∗*^
Exercise	0.339	(0.138, 0.832)	0.0180^*∗*^
Do you have full access to basic needs (water, food, shelter, electricity, etc.)?	0.264	(0.117, 0.594)	0.0010^*∗*^
Less than $50 income	5.972	(1.212, 29.430)	0.0280^*∗*^
Between $50 and $100 income	1.902	(0.537, 6.726)	0.3190
Between $100 and $300 income	1.763	(0.641, 4.850)	0.2720
Between $300 and $500 income	0.943	(0.319, 2.782)	0.9150
Between $500 and $1,000 income	0.751	(0.246, 2.293)	0.6140
Above $1,000 income	Reference category	Reference category	Reference category

^*∗*^Significant *P* values (*P* < 0.05).

## Data Availability

The dataset used and/or analyzed during the current study can be made available from the corresponding author upon reasonable request.
